# P-41. Effectiveness of recombinant zoster vaccine against postherpetic neuralgia in a real-world setting

**DOI:** 10.1093/ofid/ofae631.248

**Published:** 2025-01-29

**Authors:** Ousseny Zerbo, Joan Bartlett, Bruce Fireman, Kristin Goddard, Jonathan Duffy, Jason Glanz, Allison L Naleway, James Donahue, Tara C Anderson, Nicola P Klein

**Affiliations:** Division of Research Kaiser Permanente Vaccine Study Center, Oakland, CA; Kaiser Permanente Vaccine Study Center, Oakland, California; Division of Research Kaiser Permanente Vaccine Study Center, Oakland, CA; Division of Research Kaiser Permanente Vaccine Study Center, Oakland, CA; CDC, Atlanta, Georgia; Institute for Health Research, Kaiser Permanente Colorado, Denver, Colorado, Denver, Colorado; Kaiser Permanente Center for Health Research, Portland, Oregon; Marshfield Clinic Research Institute, Marshfield, Wisconsin, Marshfield, Wisconsin; Centers for Disease Control and Prevention, Atlanta, Georgia; Division of Research Kaiser Permanente Vaccine Study Center, Oakland, CA

## Abstract

**Background:**

Recombinant zoster vaccine (RZV) is highly effective in preventing herpes zoster (HZ). Effectiveness against postherpetic neuralgia (PHN) was nearly 90% in clinical trials but has not been extensively evaluated in real-world settings. The objective of this study was to evaluate real-world RZV effectiveness against PHN overall, by time-since-vaccination, by age, by corticosteroid use, and by time between the two doses.

**Methods:**

\We conducted a prospective cohort study from January 1, 2018 through December 2022 in persons aged ≥ 50 years at 4 healthcare systems in the Vaccine Safety Datalink (VSD) in the US. Using diagnosis codes, we identified PHN cases between 90 and 365 days after an incident HZ episode. We used Cox regression to compare the hazard of PHN in fully (≥30 days after dose 2) and partially (≥30 days after dose 1) vaccinated persons with unvaccinated persons, adjusted for demographics, comorbidities, prior receipt of zoster vaccine live, VSD site and calendar time. Vaccine effectiveness (VE) was calculated as one minus the adjusted hazard ratio.

**Results:**

Of the 45333 incident HZ cases, 2922 (6.4%) were diagnosed with PHN, of which 2775 were unvaccinated. The unadjusted incidence of PHN per 100000 person years was 43.2 among unvaccinated, 17.4 among partially vaccinated, and 7.8 among fully vaccinated persons. After adjustment, VE against PHN of partial vaccination was 68.9% (95% confidence interval [CI] 59.3% – 76.2%) and VE of full vaccination was 87.1% (CI 83.2% - 90.0%). VE of full vaccination was 91.1% during the first year, 89.8% during the second year, and 77.0% after the second year. Among fully vaccinated persons, VE was 93.1% among persons who received their first dose at ages 50-64 years and 85.9% among those who received their first dose at ages ≥65 years; VE was 74.9% in persons who had received corticosteroids within 90 days of vaccination compared to 88.3% in persons who had not. VE was 86.0% in persons who received their second dose within the recommended 6 months after their first dose compared with 91.4% in persons who received their second dose > 6 months.

**Conclusion:**

Two-dose VE against PHN was 87%, which was similar to that of clinical trials. One-dose VE was 69%, underscoring the importance of the second dose. Two-dose VE may have waned after 2 years.

**Disclosures:**

**Jason Glanz, PhD**, Hillevax: Grant/Research Support **Nicola P. Klein, MD, PhD**, CSL Seqirus: Grant/Research Support|GlaxoSmithKline: Grant/Research Support|Merck: Grant/Research Support|Pfizer: Grant/Research Support|Sanofi Pasteur: Grant/Research Support

